# He^+^ FIBID-fabricated 3D AFM tip architectures: an exploratory study of hollow pillars, helices, and spirals

**DOI:** 10.3762/bjnano.17.62

**Published:** 2026-07-09

**Authors:** Alba Arroyo-Fructuoso, Ana Galet, Gregor Hlawacek, Rosa Córdoba

**Affiliations:** 1 Institute of Molecular Science, Universitat de València, Catedrático José Beltrán 2, 46980 Paterna, Spain; 2 Institute for Ion Beam Physics and Materials Research, Helmholtz-Zentrum Dresden-Rossendorf, Bautzner Landstraße 400, 01324 Dresden, Germany

**Keywords:** 3D-printed tips, AFM tips, beam induced nanomanufacturing, focused ion beam induced deposition (FIBID), helium ion microscope, nanohelix, nanopillar, nanospiral, scanning tip microscopy, surface topography, tungsten carbide

## Abstract

Atomic force microscopy (AFM) relies strongly on tip geometry and mechanical integrity for stable and reproducible surface measurements. Here, we present an exploratory study of tungsten–carbon (W–C) AFM tips with complex three-dimensional (3D) architectures fabricated by helium ion beam-induced deposition (He^+^ FIBID) directly onto commercial AFM cantilevers bearing a pre-existing tip. Hollow nanopillars, nanohelices, and nanospirals were tested on a calibrated reference sample with a nominal step height of 20 nm using two AFM instruments under comparable operating conditions. Under the conditions explored here, selected 3D-printed tips reproduced the nominal step height of the calibration structure, yielding values consistent with those obtained using commercial probes on the same sample. To relate probe operation to structural outcome, each tip was examined by electron microscopy before and after AFM use. The main degradation modes were geometric deformation of the apex or shaft and mechanical fracture, most frequently at the tip–cantilever interface. While a few structures remained operational through repeated measurement cycles, others failed during the initial approach or early scanning stages, highlighting current limitations in robustness and reproducibility. These results show that complex He^+^ FIBID-grown nanoarchitectures can operate as AFM probes under basic test conditions, while also making clear that substantial optimization is still required.

## Introduction

The atomic force microscope (AFM) is a versatile technique for nanoscale surface characterisation that enables topographic measurements with sub-nanometre resolution by means of a sharp tip mounted at the end of a flexible cantilever [1]. During operation, the tip scans across the sample surface and responds to local interactions such as van der Waals, electrostatic, and short-range repulsive forces. Owing to its minimal sample preparation requirements and broad operational flexibility, AFM has become a widely used tool across chemistry, physics, materials science, nanotechnology, and the life sciences [2]. The continued diversification of AFM modes has also driven the development of specialised probes for electrical [3], thermal [4], optical [5], and magnetic [6] measurements, as well as biological characteristics [7].

In all these applications, tip geometry remains a central parameter because it directly affects access to surface features, tip–sample convolution, mechanical stability, and, ultimately, the reliability of the measured signal. For this reason, the fabrication of non-conventional AFM probes has attracted increasing interest, particularly in the context of focused-beam nanomanufacturing, where the geometry and material composition of the probe can be tailored beyond the constraints of standard conical or pyramidal commercial tips. Among direct-write methods, focused ion beam-induced deposition (FIBID) [8–9] and focused electron beam-induced deposition (FEBID) [10–11] provide a route to grow three-dimensional (3D) nanoarchitectures with high spatial control. In these approaches, precursor molecules are adsorbed to the substrate surface, where local beam irradiation induces their dissociation. The nonvolatile products remain at the irradiated region and accumulate to form the deposit, while volatile fragments are removed by the vacuum system. Such methods have enabled the direct fabrication of functional nanostructures on a range of substrates, including AFM cantilevers.

FEBID and FIBID have already been used to fabricate AFM nanopillar-type tips, most commonly from carbon- [12], cobalt- [13], iron- [14], platinum- [15–19], or tungsten-based precursors [20–21]. The resulting probes can be attractive because they allow for site-specific growth directly on the cantilever and can, in principle, combine tailored geometry with functional material properties. For instance, cobalt- and iron-based deposits exhibit high purity [22–23], making them suitable candidates for magnetic force microscopy (MFM) tips [13–14]. However, the magnetic performance of cobalt-based deposits may be affected by oxidation under ambient conditions, as the formation of cobalt oxide can reduce or suppress ferromagnetic behaviour. In contrast, the platinum–carbon deposits produced via FEBID exhibit a high carbon content and contain embedded platinum grains measuring approximately 3–5 nm in diameter [24]. This high carbon content results in elevated electrical resistivity [15], yet such deposits have demonstrated utility in diverse applications, including micro- and nanoelectromechanical systems (MEMS/NEMS) [16], nanothermistors for thermal sensing [25], and electrical characterisation studies [15,17]. To mitigate carbon contamination in FEBID-grown structures, various post-deposition purification strategies have been developed, including both in situ and ex situ approaches [22,24,26–31]. While these methods can significantly improve material purity and functional properties, their application to AFM tips requires particular consideration.

In tip-based applications, post-deposition treatments may alter the geometry of the structure, especially at the apex region, which is critical for imaging performance. Changes in tip radius, shape, or structural integrity can directly affect lateral resolution and tip–sample interaction. Therefore, although purification strategies are well established for planar nanostructures, their implementation in AFM tips must balance improvements in material properties with the preservation of the original nanoscale geometry.

For high-aspect-ratio AFM imaging, carbon nanotube (CNT) tips remain an important benchmark because of their small effective radii, flexibility, and long-established use in confined or deep-feature imaging [32–34]. In comparison, direct-write approaches such as FIBID and FEBID are not necessarily expected to outperform CNT or commercial ultrasharp probes in conventional topographic imaging. Their main appeal lies instead in deterministic on-cantilever growth, geometric freedom, and the possibility of integrating functional materials and non-standard architectures that would be difficult to realise by conventional fabrication routes.

Within FIBID, helium ion beam-induced deposition (He^+^ FIBID) is particularly attractive for the fabrication of 3D nanostructures because of the small beam diameter and the growth conditions that favour narrow, high-aspect-ratio deposits [18,21]. Previous studies have shown that He^+^ FIBID can produce W–C nanoarchitectures with relatively high metal content, crystalline character, and conductive behaviour, including hollow nanopillars and nanohelices [35–37]. These structures are therefore interesting not only from a fabrication point of view, but also as candidate building blocks for future multifunctional scanning probes.

The aim of the present work is to use He^+^ FIBID as a 3D nanoprinting route to fabricate several complex tip architectures directly on AFM cantilevers and to address a relevant question: Can these structures operate as AFM probes under basic test conditions, and what do the failure modes of the majority of structures, and the limited operational success of a few, reveal about the practical constraints of this approach?

To address this question, we fabricated W–C hollow nanopillars, nanohelices, and nanospirals onto commercial AFM cantilevers and tested them on a standard calibration sample with a nominal 20 nm step height. Height values were extracted from AFM topographs and checked against measurements from commercial reference probes serving as a step height baseline. Importantly, each printed structure was also inspected by electron microscopy before and after AFM use in order to document morphology changes, degradation pathways and architecture-dependent failure modes. In this way, the work is framed as an exploratory and limitations-aware study that combines fabrication, basic operation, and post-operation structural auditing to identify both the current shortcomings and the design lessons that can inform future 3D nanofabricated AFM probes.

## Results and Discussion

### 3D nanoprinting of AFM tips

**Figure 1:**
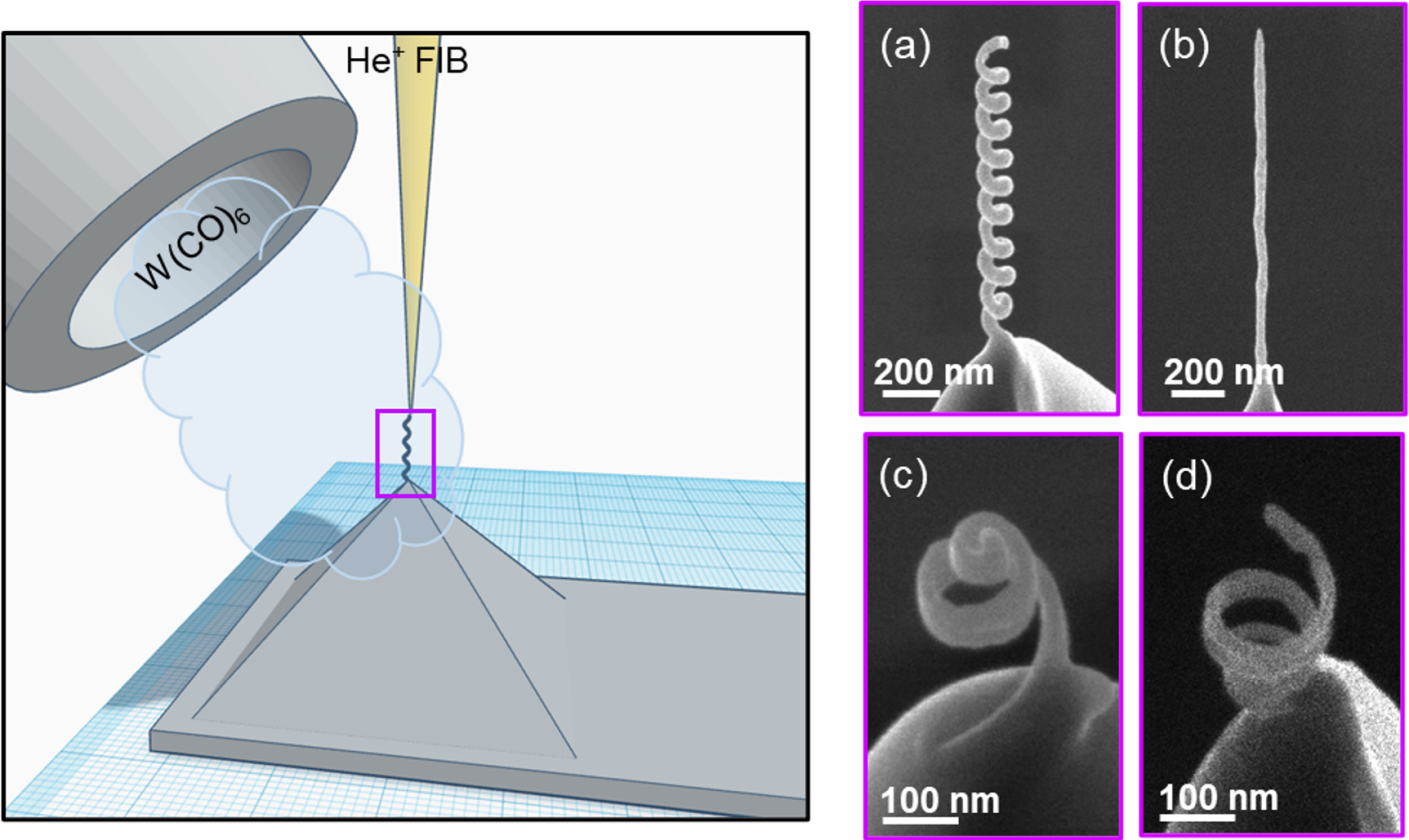
Schematics showing the 3D-printed nanoarchitectures fabricated by He^+^ FIBID onto commercial AFM cantilevers: (a) nanohelix, (b) hollow nanopillar, (c) inward Archimedean nanospiral, and (d) outward Archimedean nanospiral. The corresponding HIM images of the fabricated nanoarchitectures are shown in subfigures (a–d).

The 3D-printed nanoarchitectures investigated as functional AFM tips included nanohelices, hollow nanopillars, and nanospirals. These nanoarchitectures were directly fabricated onto tips attached to commercial AFM cantilevers bearing a commercial tip for practical reasons related to alignment, fabrication control, and AFM handling, as well as to enable direct comparison with standard cantilevers. The 3D nanoprinted tips were grown using He^+^ FIBID, as depicted in Figure 1. To preserve the original cantilever–tip geometry during AFM operation, the printed structures were fabricated with the same inclination as the commercial tip, maintaining the nominal cantilever-to-sample tilt angle of approximately 10–15° typically used in AFM systems.

The fabrication was performed using the ZEISS ORION NanoFab system, equipped with a single-needle, multiprecursor gas injection module (Oxford Instruments OmniGIS II), which enabled precise delivery of precursor gases into the processing chamber. To ensure reproducibility and structural fidelity, the He^+^ FIBID process was governed by the following fabrication parameters: tungsten hexacarbonyl as precursor material, W(CO)_6_; *T*_precursor_ = 55 °C; GIS_needle diameter_ ≈ 500 μm; GIS*_z_* ≈ 500 μm; GIS*_x,y_* ≈ 60 μm; *P*_base_ ≈ 1.5 × 10^−7^ mbar; *P*_process_ ≈ 5.2 × 10^−6^ mbar; acceleration voltage = 30 kV; ion beam current ranging from 0.96 pA to 1.07 pA.

Each type of nanoarchitecture was fabricated following distinct patterning protocols tailored to its geometry: (1) Nanohelices were patterned in circumference mode, with a fixed nanowire diameter of 75 nm, a step size of 10 nm, a dwell time of 500 ms, and loop counts ranging from 9 to 15, resulting in total processing times of 60 or 180 s. (2) Hollow nanopillars were written in spot mode with total processing times of 30 or 45 s, yielding outer diameters of ≈45 nm and inner diameters of ≈6 nm. (3) Nanospirals were generated from node lists based on Archimedean spirals, with diameters of 150 or 200 nm, a step size of 5 nm, a dwell time of 120 ms, and total processing times of ≈20 s

To further investigate the practical suitability of these novel nanoarchitectures as AFM tips, a diverse set of tips was fabricated with controlled dimensional variations: five nanohelices (H1–H5), six hollow nanopillars (P1–P6), and seven nanospirals (S1–S7) (Tables 1–3). Detailed dimensions for each tip are provided in Supporting Information File 1.

**Table 1:** Dimensional characterisation of nanohelix tips fabricated onto commercial AFM cantilevers using He^+^ FIBID, highlighting variations in growth direction, height, and loop count. The nominal nanohelix diameter (*d*_NH_) and nanowire diameter (*d*_NW_) remain constant at 75 nm and 50 nm, respectively.

Nanohelices	Growth direction	Height (μm)	Loops	*d*_NH_ (nm)	*d*_NW_ (nm)

H1–H5	R- or L-	0.87–1.04	9–15	75	50

**Table 2:** Dimensional characterisation of hollow nanopillar tips fabricated onto commercial AFM cantilevers using He^+^ FIBID, emphasizing changes in length while maintaining a constant outer diameter (*d*_o_) of 50 nm and inner diameter (*d*_i_) of 6 nm.

Hollow nanopillars	Length (μm)	*d*_o_ (nm)	*d*_i_ (nm)

P1–P6	0.6–3.2	50	6

**Table 3:** Dimensional characterisation of nanospiral tips fabricated onto commercial AFM cantilevers using He^+^ FIBID, indicating the growth pattern, height, number of loops, and nanospiral diameter (*d*_spiral_). The nanowire diameter (*d*_NW_) was kept constant at 30 nm.

Nanospirals	Growth pattern	Height (nm)	Loops	*d*_spiral_ (nm) max/min

S1–S7	inward or outward	150–220	1–3	50–257

The formation mechanism of hollow nanostructures during He^+^ FIBID has been reported previously [35]. In this growth mode, the interplay between precursor deposition and ion-beam-induced milling promotes the formation of a central cavity during deposition. The inner and outer diameters of the tubular structures can be tuned through ion beam current and dose, and a progressive tapering towards the tip end reduces the effective tip radius. For the purposes of the present work, this prior knowledge supports the designation of the printed structures as hollow nanopillars.

Commercial AFM tips with standard and ultrasharp geometries were included as reference probes in order to verify whether the 3D-printed nanoarchitectures could perform basic topographic measurements on the selected calibration sample under comparable operating conditions. Their role in this study is strictly comparative and practical: They provide a reference for the nominal step height on the chosen sample.

The printed nanoarchitectures display markedly different geometrical features, including variable heights, opening angles, and apex radii, depending on the design. Nanohelix tips exhibit heights ranging from 0.47 to 1.04 μm and an approximate tip radius of 23 nm. Nanopillar structures span a broader height range, from 0.22 to 3.19 μm, with a similar tip radius of about 23 nm. Nanospiral tips are shorter, with heights between 0.05 and 0.22 μm, and exhibit a smaller approximate tip radius of 15 nm. Because of the observed variability among nominally similar structures and the post-operation changes documented by SEM, any apparent relation between geometry and AFM behaviour should be interpreted as qualitative and exploratory rather than conclusive.

With these geometries established, we adopted a two-stage workflow to assess the eighteen printed tips. In the first stage, each tip was mounted in the AFM and used to scan a calibrated reference surface over a total area of 52 μm^2^ (two scans of 25 μm^2^ and two of 1 μm^2^). This stage was intended only to establish basic operation on a standard sample and to extract step height values under matched conditions. In the second stage, the same tips were transferred to SEM in order to document their post-use condition. This before/after workflow allowed us to relate apparent AFM functionality to the actual post-operation morphology of the structures.

**Figure 2:**
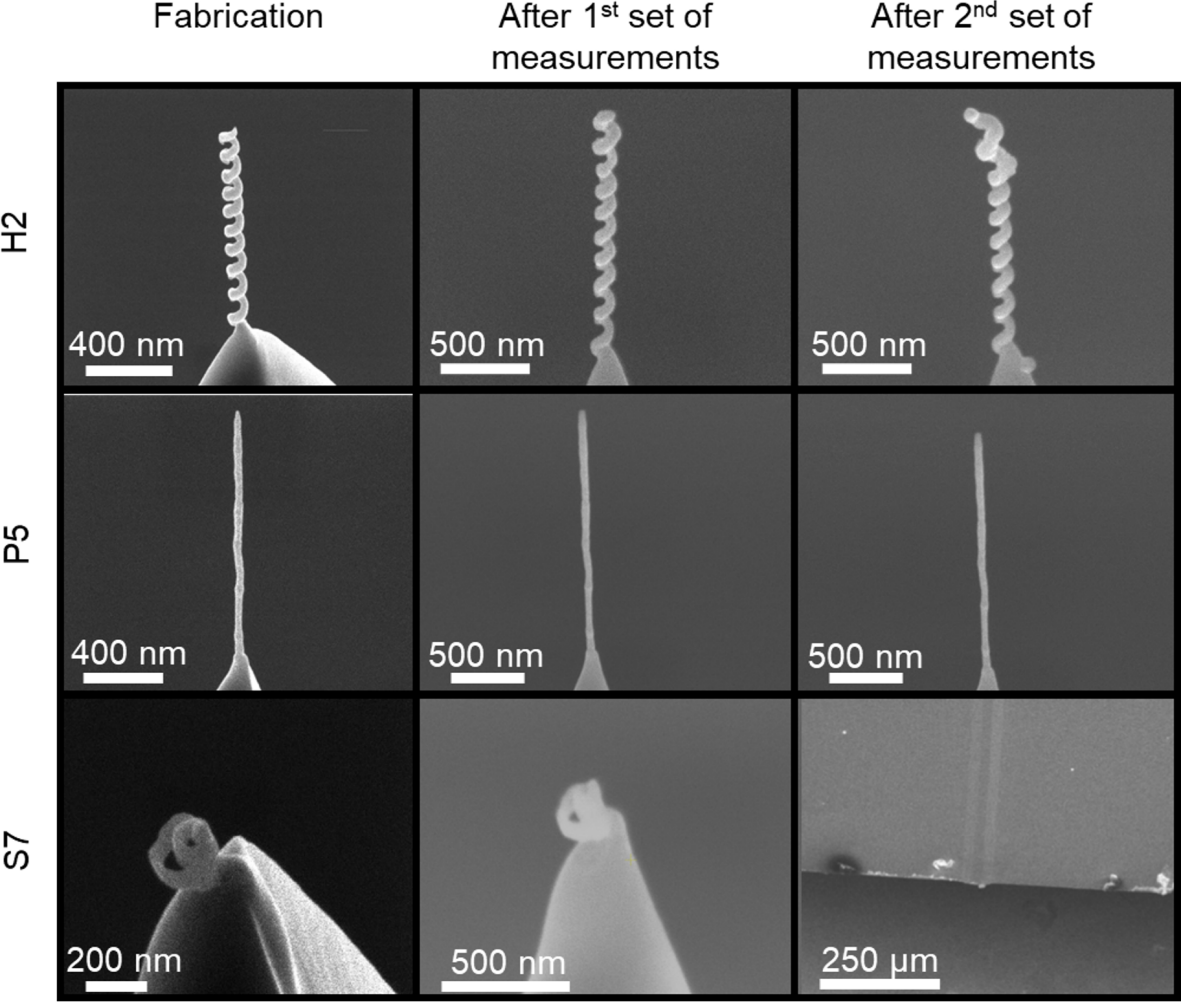
First column: HIM images of the as-fabricated 3D-printed tips with nanohelix, nanopillar, and nanospiral geometries, acquired using an ORION NanoFab microscope at a 45° tilt angle. Second and third columns: SEM images of the same tips after the first and second AFM measurement cycles, respectively, acquired using a Thermo Fisher SCIOS 2 microscope at a 52° tilt angle.

From this point on, we focus on H2 and P5, which preserved their structural integrity through two AFM/SEM cycles, and on S7, which remained operational during the AFM measurements but was unavailable for the second SEM inspection because the cantilever was lost during subsequent SEM sample preparation (Figure 2). The remaining fifteen tips exhibited SEM-visible damage immediately after the first AFM campaign. Representative failure cases are shown in Supporting Information File 1.

### Topographic characterisation

**Figure 3:**
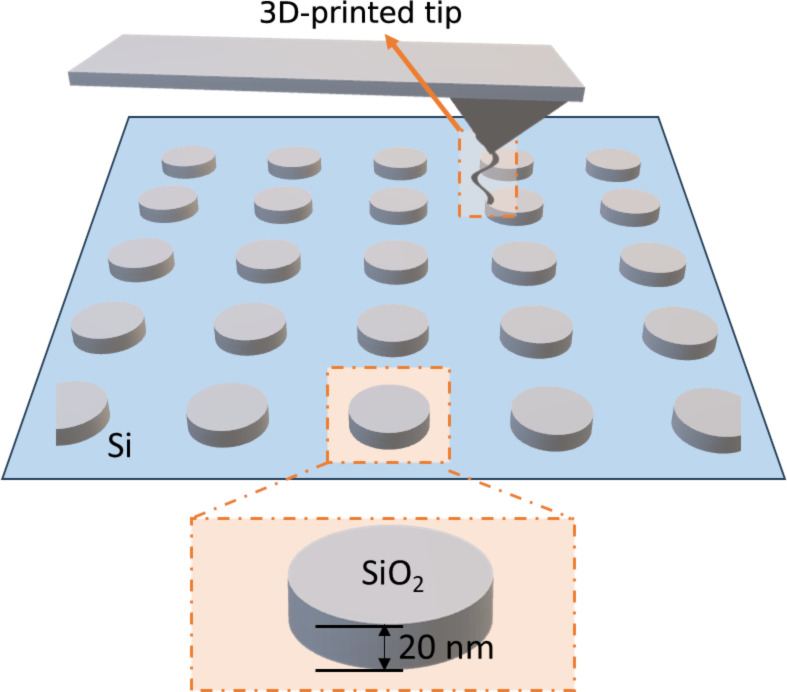
Topographic sketch of the silicon pattern employed in AFM measurements. A circular feature with a nominal height of 20 nm and a diameter of 3.3 μm is highlighted to mark the reference structure employed for performance evaluation.

To evaluate the basic operation of the 3D-printed tips, AFM measurements were carried out on a TGXYZ01 commercial Si wafer featuring raised SiO_2_ discs with a nominal height of 20 nm (2% accuracy) and a diameter of 3.3 μm (Figure 3). All probes were tested under matched scanning conditions.

**Figure 4:**
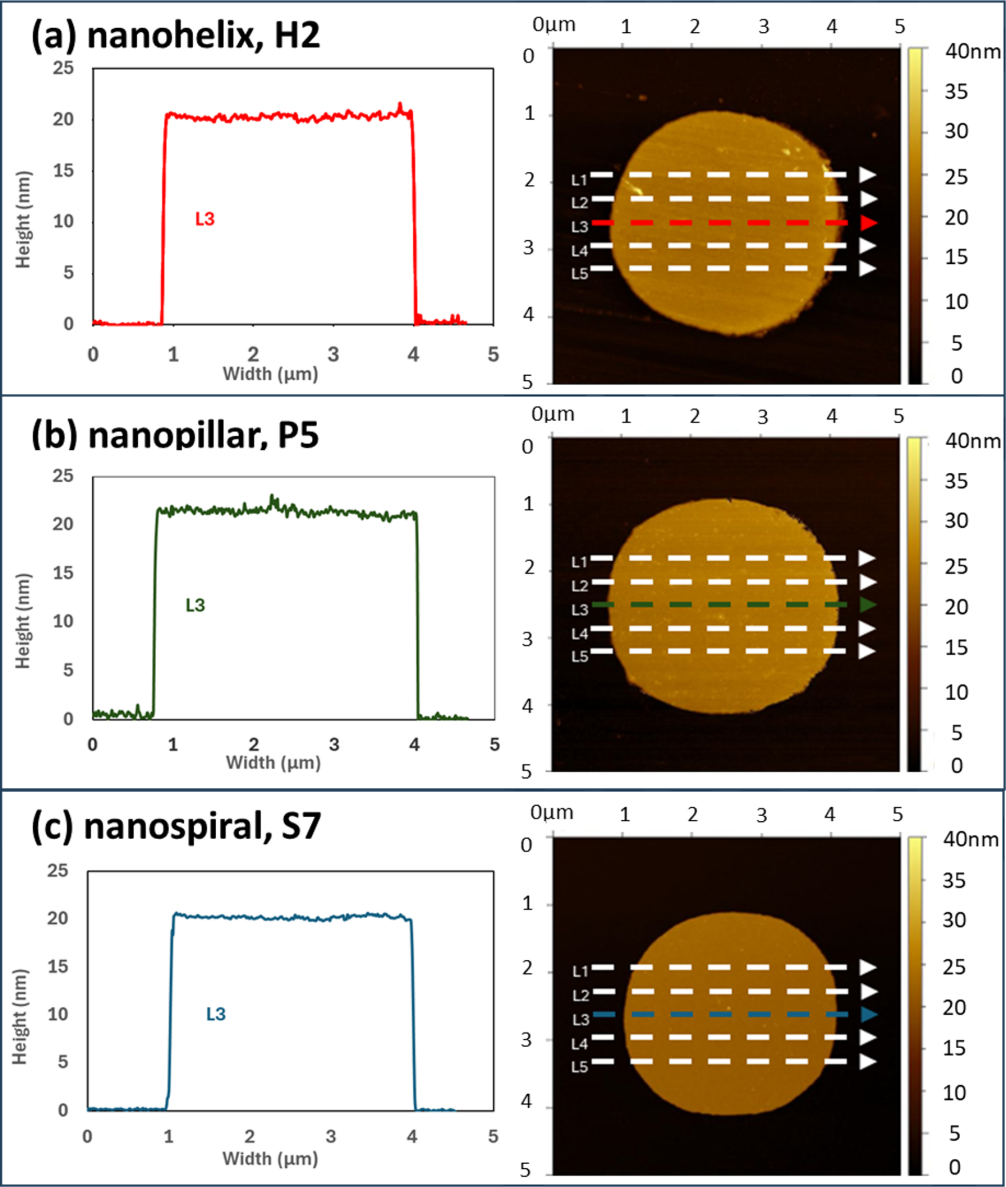
Representative 2D AFM images and one of the five extracted height profiles for measurements obtained using (a) nanohelix H2, (b) nanopillar P5, and (c) nanospiral S7. Local peaks visible in the AFM images may correspond to contamination particles or pattern defects.

Topographic characterisation was performed using two AFM instruments, a Veeco Multimode NanoScope V and a Dimension Icon (Bruker), in tapping mode. Before approaching the surface, the cantilevers were tuned to identify their resonance frequency and to adjust the driving frequency and oscillation amplitude for stable tapping-mode operation. Scans were recorded over areas of 25 μm^2^ and 1 μm^2^, using 512 lines and a scan rate of 0.5 Hz. The AFM topographs were processed with Gwyddion v2.65 using a consistent workflow that included mean-plane levelling, row alignment, and tilt correction. Representative profiles for the selected structures H2, P5, and S7 are shown in Figure 4.

For the three structures that completed the measurement campaign without SEM-visible damage, step height values extracted from five parallel height profiles over 25 μm^2^ scan areas were 21.6 ± 0.7 nm (H2), 21.5 ± 0.5 nm (P5), and 20.5 ± 0.2 nm (S7), consistent with those obtained using the commercial reference probes (20.9 ± 0.2 nm and 21.0 ± 0.3 nm) and with the nominal 20 nm value. These measurements confirm that the three surviving structures can reproduce the step height of the calibration feature under the tested conditions, but do not constitute a performance benchmark relative to commercial tips. No surface roughness analysis is reported. With effective tip radii of approximately 15–23 nm and a calibration surface that is essentially flat between the patterned features, any roughness values extracted from these topographs would primarily reflect tip–sample convolution, instrument noise, and drift rather than true surface texture. A meaningful roughness analysis would therefore require both a purpose-designed reference sample and a statistically adequate set of probes with well-characterised apex geometry, which are beyond the scope of the present study. The three working probes produced qualitatively good AFM images. For obtaining quantitative performance values, a more comprehensive AFM benchmarking study would require dedicated reference samples (e.g., tip characterizers) and a statistically broader set of optimised probes. However, given the mechanical instability of the investigated probe geometries such a study is neither feasible nor useful.

### Evaluation of 3D-printed tips after topographic characterisation

The structural integrity and wear of the W–C 3D-printed tips were evaluated by SEM before AFM operation and after the first and second measurement cycles. The analysis covered all investigated nanoarchitectures together with the two commercial reference tips, allowing the morphological evolution of the structures under repeated operational stress to be monitored. SEM images acquired before and after AFM measurements for all investigated tips are provided in Supporting Information File 1.

The different nanoarchitectures exhibited distinct degradation behaviours. Hollow nanopillars showed a higher tendency to fracture during the initial approach, consistent with the larger bending moments expected for slender vertical structures. Nanospiral tips were more susceptible to progressive geometric deformation. Nanohelices showed mixed behaviour, with some structures remaining usable while others were damaged early in the measurement campaign. These differences indicate that nanoarchitecture influences the mechanical response of the probes during AFM operation [38].

In most cases, failure occurred at or near the tip–cantilever interface, indicating that this region is the main structural weak point of the printed nanoarchitectures. Even the commercial reference probes showed some apex blunting after repeated use, as documented in Supporting Information File 1. It is also important to note that not all observed damage can be attributed solely to intrinsic geometric fragility. As detailed in Supporting Information File 1, a subset of early failures showed local apex melting consistent with electrical-discharge events associated with insufficient AFM grounding identified during the measurement campaign. These cases should be distinguished from purely mechanical failure modes. Even with this caveat, the overall picture remains clear: The present tip designs show limited robustness and limited reproducibility under the tested conditions.

The frequent occurrence of fracture at the base of the deposit underscores the need to optimise the mechanical design of the tip–cantilever interface. This limitation is consistent with behaviour reported for other high-aspect-ratio probes.

Several design modifications can be considered to reduce stress concentration at the anchoring region. Decreasing the effective tip length would reduce the bending moment experienced during approach and scanning, thereby lowering the probability of mechanical failure. Reinforcing the base through tailored growth protocols, for example, by increasing the local thickness or by incorporating supporting geometries such as truncated-cone profiles or lateral buttresses, could promote a more homogeneous stress distribution and enhance structural stiffness. Smoothing the geometric transition between the tip and the cantilever, for example, by avoiding abrupt variations in cross section, may further reduce stress concentrators. More generally, architecture-specific parameters such as wall thickness, pitch, local support features, and final apex termination are likely to be important variables in future optimisation.

## Conclusion

We fabricated eighteen AFM tips with hollow nanopillar, nanohelix, and nanospiral architectures by He^+^ FIBID and evaluated them under basic imaging conditions on a calibrated reference sample. Fifteen of the eighteen printed structures exhibited SEM-visible damage during or immediately after the first measurement cycle, revealing the limited mechanical robustness of the current designs. Of the three remaining structures, two (H2 and P5) preserved their structural integrity through two consecutive AFM/SEM cycles; the third (S7) remained operational during AFM measurements but the cantilever was subsequently lost during SEM sample preparation. These selected structures reproduced the nominal step height of the calibration feature under the tested conditions, confirming basic probe functionality, but without demonstrating any imaging advantage over commercial tips.

The systematic before/after electron microscopy inspection shows that the dominant degradation modes are apex or shaft deformation and mechanical fracture, most frequently at the tip–cantilever interface. This region is the principal structural weak point of the current designs.

The primary contribution of this work is a failure-mode analysis that identifies where and how the current He^+^ FIBID tip architectures break down. By combining direct-write fabrication, basic AFM operation on a calibration surface, and post-operation structural inspection, the study identifies the design parameters most in need of optimisation, that is, reducing effective tip length to lower the bending moment at the anchoring region, reinforcing the base through truncated-cone profiles or lateral buttresses, smoothing abrupt cross-sectional transitions, and tuning architecture-specific variables such as pitch, wall thickness, and apex termination. None of these modifications were implemented in the current study; they define the essential scope of future work before complex He^+^ FIBID architectures can be used as reliable AFM probes.

Beyond topographic operation, the conductive character of W–C He^+^ FIBID deposits and the geometric flexibility of the deposition method may make such structures attractive for future multifunctional or application-specific probes. These possibilities remain prospective and will require both substantially improved mechanical robustness and dedicated functional characterisation.

## Supporting Information

File 1 Additional figures and tables.

## Data Availability

Data generated and analyzed during this study is available from the corresponding author upon reasonable request.
