# Supplementary material for: He+ FIBID-fabricated 3D AFM tip architectures: an exploratory study of hollow pillars, helices, and spirals
Source: Beilstein J Nanotechnol. 2026 Jul 9;17:863–71. doi: 10.3762/bjnano.17.62 (PMC13358899; doi:10.3762/bjnano.17.62)
Supplement: File 1 — Additional figures and tables. [file Beilstein_J_Nanotechnol-17-863-s001.pdf]

BEILSTEIN JOURNAL OF NANOTECHNOLOGY

Supporting Information

for

He$^+$ FIBID-fabricated 3D AFM tip architectures: an exploratory study of hollow pillars, helices, and spirals

Alba Arroyo-Fructuoso, Ana Galet, Gregor Hlawacek and Rosa Córdoba

Beilstein J. Nanotechnol. **2026,** *17,* 863–871. doi:10.3762/bjnano.17.62

Additional figures and tables

License and Terms: This is a supporting information file under the terms of the Creative Commons Attribution License (https://creativecommons.org/licenses/by/4.0). Please note that the reuse, redistribution and reproduction in particular requires that the author(s) and source are credited and that individual graphics may be subject to special legal provisions.
The license is subject to the *Beilstein Journal of Nanotechnology* terms and conditions: (https://www.beilstein-journals.org/bjnano/terms)

1. 3D-PRINTED TIP IMAGES

SEM inspection uncovered the partial or complete burning of the apex due to electrical discharge. In total, four printed tips suffered this damage, which was traced to an insufficient grounding of the AFM system identified early in the campaign; the affected tips showed a molten droplet at the apex. Burning events occurred predominantly in nanohelix tips, coinciding with the grounding issue and therefore not necessarily reflecting an intrinsic weakness of that nanoarchitecture.

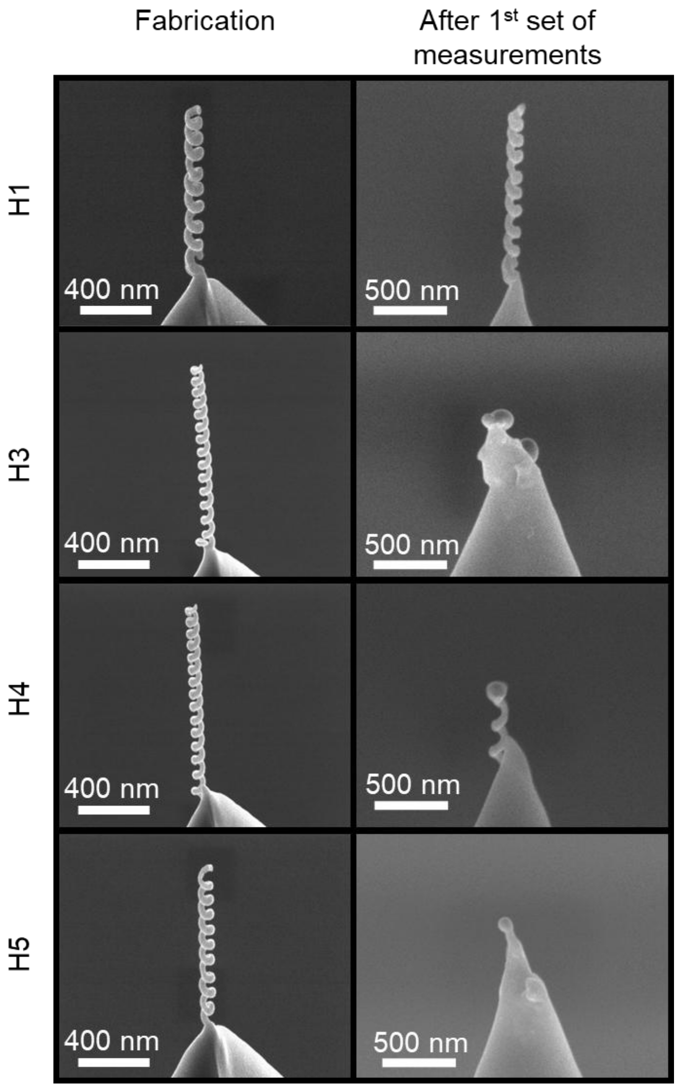

Figure S1: HIM and SEM images illustrate the tips with nanohelix architecture fabricated by He^{+} FIBID, shown both after fabrication and after the first set of measurements, for those tips that did not preserve their physical integrity through two consecutive AFM/SEM cycles.

SEM inspection of the hollow nanopillar tips shows that this nanoarchitecture most frequently fractured during the AFM approach. In some cases, electrical-discharge damage, attributable to insufficient AFM grounding was also evident, leaving a molten droplet at the apex.

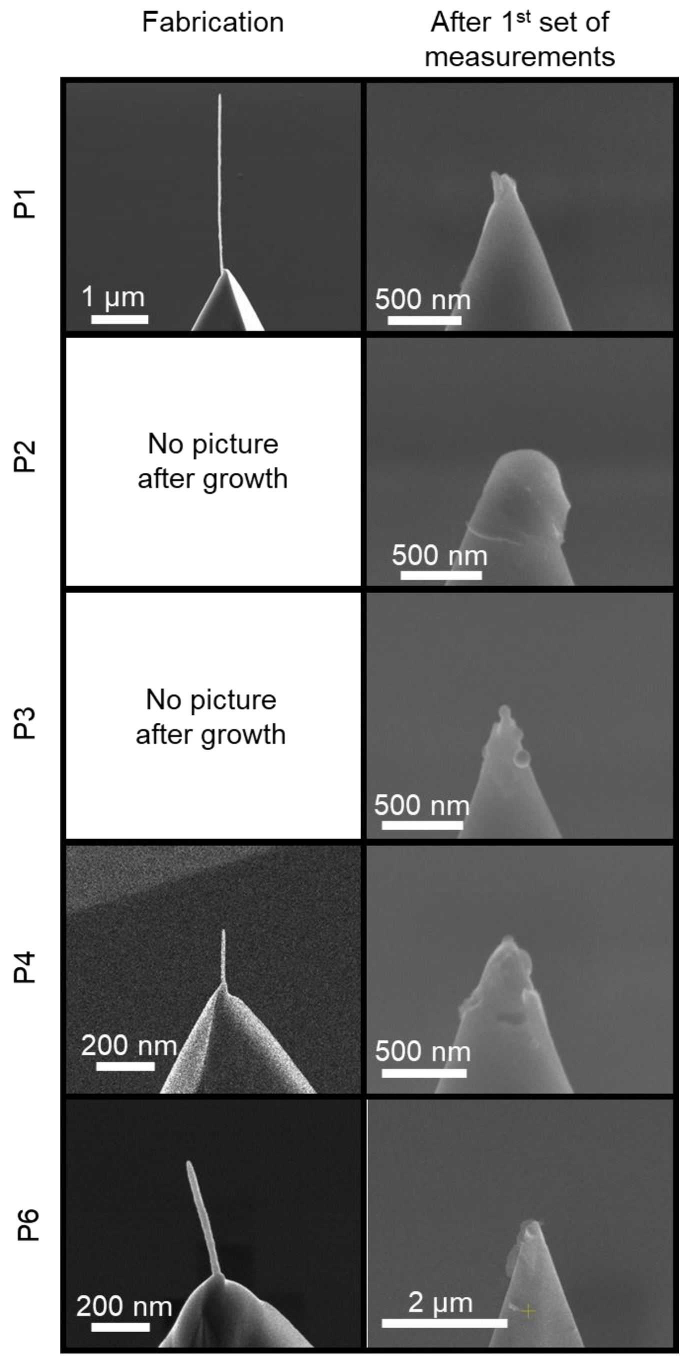

Figure S2: HIM and SEM images illustrate tips with nanopillar architecture fabricated by He^+ FIBID, shown both after fabrication and after the first set of measurements, for those tips that did not preserve their physical integrity through two consecutive AFM/SEM cycles.

Nanospiral tips are markedly more susceptible to mechanical failure via structural deformation.

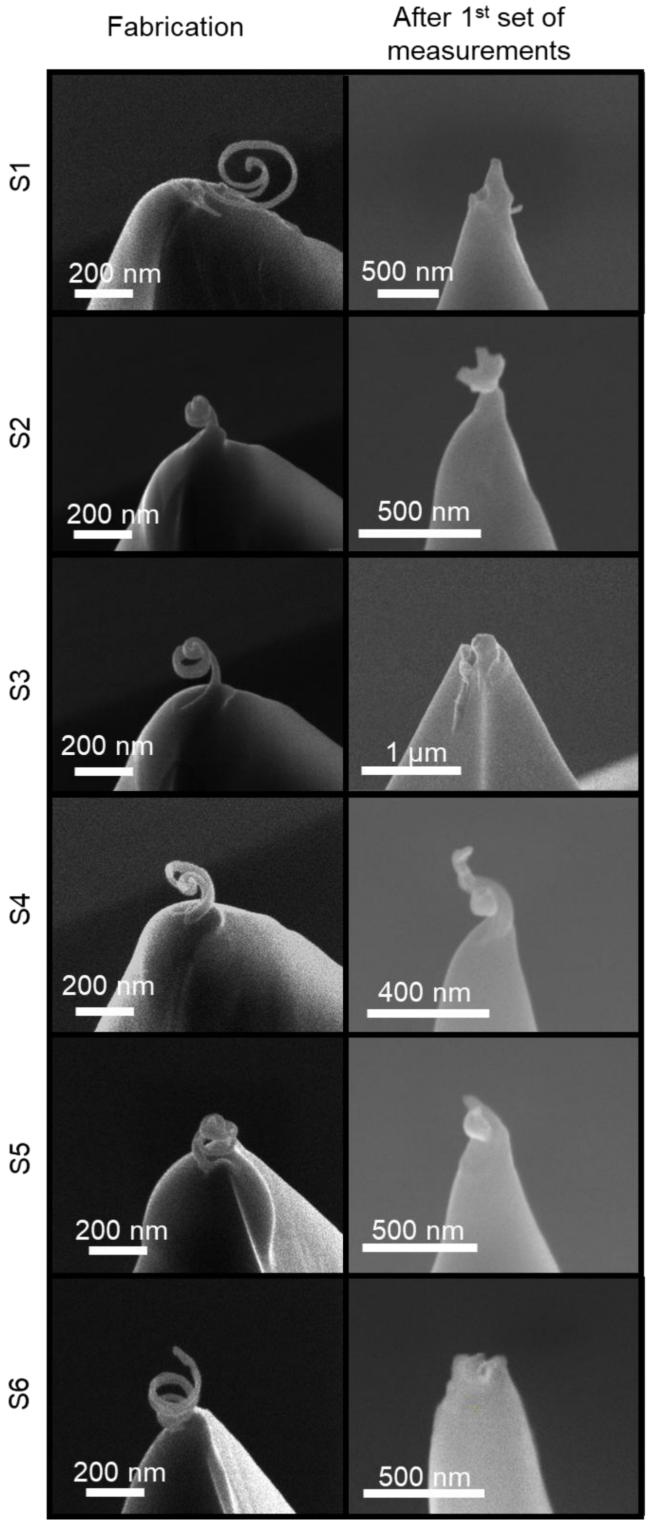

Figure S3: HIM and SEM images illustrate tips with nanospiral architecture fabricated by He^{+} FIBID, shown both after fabrication and after the first set of measurements, for those tips that did not preserve their physical integrity through two consecutive AFM/SEM cycles.

The SEM images of the pristine AFM tips – used as reference standards for comparing height and roughness measurements with those obtained using the 3D-printed tips – show slight modifications at the tip apex after the first and second AFM scanning cycles, a behaviour that can occur even in tips generally considered mechanically robust.

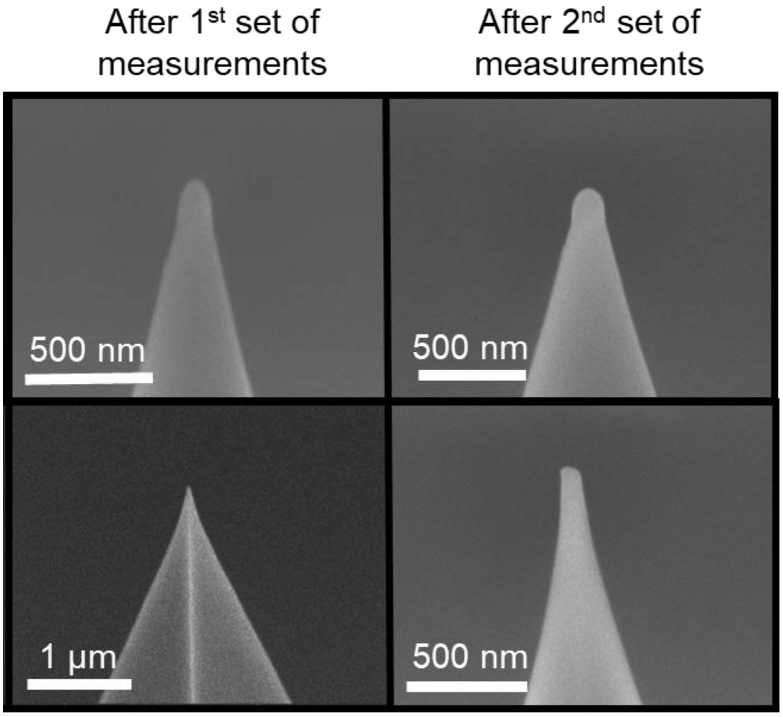

Figure S4: SEM image of the two commercial AFM reference tips—TAP 300G (standard) and RTESPA 150 (ultra-sharp)—characterised using the same protocol applied to the 3D-printed tips.

2. 3D-PRINTED TIP DIMENSIONS

Table S1: Description of nanohelix tips parameters, including modifications in growth direction, height, number of loops, length, nanohelix diameter (d_{NH} = 75 nm) and nanowire diameter (d_{NW} = 50 nm) of each nanohelix tip. Error was calculated as deviation from the average of three measurements.

Nanohelix tips	Growth	Average Height (µm)	Loops	Length (µm)
H1	Right	0.93 ± 0.02	9	2.94
H2	Left	0.89 ± 0.02	10	3.25
H3	Left	0.99 ± 0.01	14	4.50
H4	Left	1.04 ± 0.02	15	4.80
H5	Right	0.87 ± 0.02	10	3.25

Table S2: Variations in hollow nanopillar tips dimensions, focusing on length while maintaining a consistent outer (d_o = 50 nm) and inner diameter (d_i = 6 nm).

Nanopillar tips	Average Length (µm)
P1	3.19 ± 0.04
P2	2.88 ± 0.33
P3	2.30 ± 0.26
P4	0.58 ± 0.20
P5	1.24 ± 0.04
P6	0.63 ± 0.10

Table S3: Nanospiral tips dimensions indicating the growth pattern, height, number of loops, estimated length, maximum and minimum diameter of the nanospirals (d_{NS}) and the diameter of the nanowire (d_{NW} = 30 nm). Error was calculated as deviation from the average of three measurements.

Nanospiral tips	Growth	Average Height (nm)	Loops	Length (µm)	Max d_{NS} (nm)	Min d_{NS} (nm)
S1	Out	162 ± 34	2	0.94	257 ± 5	60 ± 1
S2	In	152 ± 4	3	0.52	106 ± 4	70 ± 3
S3	In	202 ± 26	2	0.40	131 ± 8	50 ± 2
S4	Out	207 ± 17	1	0.51	146 ± 4	50 ± 5
S5	In	164 ± 13	3	0.75	164 ± 13	90 ± 4
S6	Out	218 ± 16	2.5	0.91	183 ± 10	110 ± 4
S7	In	202 ± 15	2	1.04	186 ± 11	130 ± 2

3. 3D-PRINTED TIP HEIGHT AND ROUGHNESS

3.1. HEIGHT AND ROUGHNESS

Table S4: Average height and roughness from type-nanoarchitecture tips profiles obtained in the first set of measurements in the scanned regions of 25 μm². The error in height was calculated as deviation from the average of five profile measurements, and similarly, the roughness error was calculated from the average of three roughness measurements using the Gwyddion software.

Nanoarchitecture	Height (nm)	Roughness (nm)
Standard	20.9 ± 0.2	0.60 ± 0.10
Ultra-sharp	21.0 ± 0.3	0.94 ± 0.10
H1	20.3 ± 0.1	0.55 ± 0.10
H3	22.2 ± 0.7	1.68 ± 0.10
H4	20.1 ± 0.4	1.06 ± 0.10
H5	21.4 ± 0.1	0.38 ± 0.10
P1	20.8 ± 0.2	0.59 ± 0.04
P3	20.6 ± 0.1	0.57 ± 0.05
P4	21.6 ± 0.5	0.38 ± 0.08
P6	20.6 ± 0.6	0.95 ± 0.10
S1	21.2 ± 0.9	1.10 ± 0.03
S2	20.7 ± 0.3	0.83 ± 0.15
S3	21.6 ± 0.4	1.07 ± 0.04
S4	21.3 ± 0.3	0.57 ± 0.05
S5	20.8 ± 0.4	0.94 ± 0.04
S6	20.6 ± 0.4	0.53 ± 0.10
